# Thermoplastics for Clear Aligners: A Review

**DOI:** 10.3390/polym17121681

**Published:** 2025-06-17

**Authors:** José Ignacio Delgado, Pablo Kehyaian, Juan P. Fernández-Blázquez

**Affiliations:** 1 Secret Aligner S.L., C/ Sangenjo 34, 28034 Madrid, Spain; ignacio.delgado@imdea.org (J.I.D.); pablo.k@secretaligner.com (P.K.); 2 IMDEA Materials Institute, C/Eric Kandel, 2, 28906 Getafe, Spain

**Keywords:** aligners, thermoplastic, dental, force

## Abstract

With the worldwide spread of clear aligner treatment (CAT), a plethora of new thermoplastics is currently commercially available on the market, claiming to have superior properties and greater comfort. This review aims to summarise the properties of the materials and their effects on treatment effectiveness and comfort to ease material selection and also incorporate new emerging trends such as shape memory polymers (SMPs) and direct 3D printer aligners. First, a concise historical overview of orthodontics will be presented, along with the basic properties of thermoplastics and their importance in treatment. Following the individual properties, we present an analysis of optical, biocompatibility, and toxicity aspects, passing through others such as thermal, mechanical, and special methods to simulate in vivo measurements. We end with the impact of this technique on the environment and the advances and perspectives of CAT.

## 1. Introduction

Humans have been coping with dental problems since their origins, from the first dental appliances used by the Etruscans and Egyptians in 1000 BC to prevent tooth crowding, made out of gold and silver, directly attached to the dentition. The same approach remains applicable in modern metal fixed appliances made out of austenitic steels and nickel–titanium superelastic alloys (NiTi) bonded to the tooth using UV curing adhesives [1,2,3,4].

In 1923, Remensnyder introduced a new technique based on a rubber appliance that gently massaged the gingiva to ameliorate gingivitis and periodontitis symptoms [5,6]. The idea was further improved by Dr. Kensling in 1946 [7], inventing the first tooth positioner. Kensling’s invention was similar to the current way of working with the clear aligners technique. First, a practitioner took clay impressions of the patient’s dentition to obtain a mold. The mold was used to set the final desired position of the tooth and was finally pressed against a soft-vulcanized rubber to fabricate the device. Since only the final position is set, the devices only worked for small corrections and were rather uncomfortable due to their huge thickness and poor organoleptic properties.

In 1971, Robert J. Ponitz replaced the rubber for the first time with a transparent thermoplastic formed using heat and a vacuum over oral clay impressions. On some occasions, some corrections were carried out in steps, but this required concise planning and several visits to the doctor, so in general, the devices were only able to treat small misfits or to keep the oral position, and the prices were far from accessible for the general public. Thus, Ponitz’s appliances were used more as sports guards for top-performance athletes [8].

**Figure 1:**
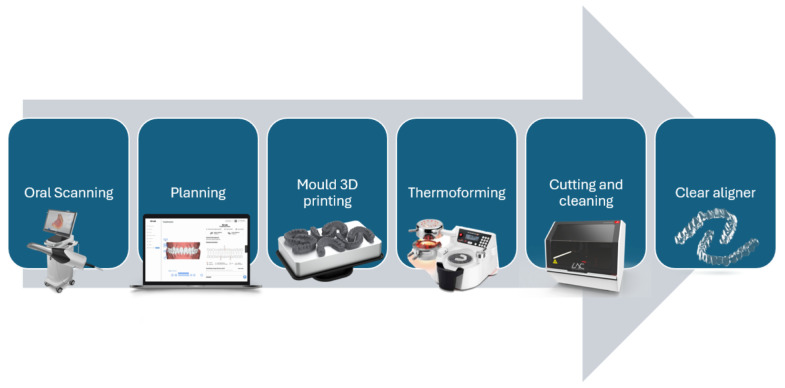
Clear aligner manufacturing process.

The final major improvement that led to CAT happened in 1997 when two Stanford students took advantage of the new CAM/CAT software that was under development at the university [9]. The technique substituted the clay impressions for CAM images taken from the oral cavity with an oral scanner, which is the most common work frame nowadays. Afterward, aided by software, the professional plans the movements, producing a digital 3D image of each step, which is sent to a 3D printer. Once all the models are printed, a thermoplastic sheet is thermoformed using heat and a vacuum over each model, and then the thermoplastic models are demolded, cut, trimmed, cleaned, sterilized, and handed to the patient as we can see in Figure 1.

We are now in the first stages of another revolution for orthodontics based on more advanced polymers with greater effectiveness and specificity in terms of patient and movement variables. Thus, we aim to give a descriptive overview of the properties of CAT plastics to choose the correct one for each situation. Moreover, 3D printed aligners and shape memory polymers are starting to appear on the market. Thus, we considered this a perfect time to review the successes and findings in the literature and also the new trends and goals of CAT.

## 2. Thermoplastics

The most common thermoplastic in CAT is polyethene terephthalate (PET), or its amorphous version, copolymerized with ethylene glycol (PETG), both of which are polyesters. Recently, other copolyesters have appeared on the market, having enhanced properties, but they are still not of extended use due to their low availability and higher price, as well as a lack of knowledge [10,11,12].

The second most common plastic group is thermoplastic polyurethane (TPU), a copolymer of hard and soft blocks joined by a urethane bond. The final properties vary with the nature of the hard and soft blocks, the relative percentage, and the interactions among them [13]. Generally, in CAT, only high-hardness TPU (hTPU) has been used due to its resemblance (in mechanical properties) to PETG, whereas middle-hardness (mTPU) and soft-hardness (sTPU) TPU are mainly used in multilayers. From now on, we will refer to the TPU family as TPU, and the specific type will be mentioned, as previously explained. TPU is said to produce a better organoleptic feeling than polyesters in general, but its price and the difficulties in processing, handling, and storing it make it less common. However, it is also more problematic in terms of allergies and chemical staining than PETG [14].

A similar scenery depicts ethylene vinyl acetate (EVA), which is a copolymer of ethylene and vinyl acetate, producing a soft transparent elastomer, the properties of which depend on the vinyl acetate (VA) percentage against ethylene, increasing the elasticity with VA content. In contrast to TPU, EVA is a widely available and relatively cheap product that is easily processed, but its dental use has been almost relegated to sportive and bruxism mouth guards. A similar fate has transpired for Polypropylene (PP), the properties of which are considered to be too low to manufacture devices for an active role; it has the additional drawbacks of being crystalline and not transparent [12].

Another less common thermoplastic on the market is polycarbonate (PC). In the case of PC, its use is not extensive due to its high price and poor aging when worn [10,14]. Moreover, misinformation regarding its biocompatibility has affected its reputation with the general public.

Thanks to Invisalign’s patent expiration, multilayers are now available to be manufactured by other brands to further improve the efficacy of tooth movement [15,16]. This strategy is based on combining two or more plastics of the already mentioned, joining different layers of them together. This approach, although more costly, seems to be more effective and comfortable in treatment [16,17,18].

Another strategy scarcely used is to blend different plastics into a new one. Different polymers are usually non-miscible, making a product that would lie in the middle of both of the components; however, some studies have pointed to the possibility of using PC as a compatibilizer between PETG and TPU [19,20] to produce a material with enhanced stress relaxation properties when compared with PETG.

Finally, the new trends in CAT research are shape memory polymers (SMPs) and 3D printing resins. SMPs are built up by a major polymeric network that defines a stable shape and a secondary network that defines a temporary shape; thus, under certain conditions, the material can recover from a temporary to a stable shape on its own. This would facilitate feasible, more effective, and faster treatments [21,22,23].

There are two means to use SMPs in dentistry: one focused on improving treatment effectiveness and the other on reducing the number of aligners used. The first approach uses the same idea as the shape of memory metal wires already in use in dentistry. An aligner with the final shape is manufactured, and the current mouth shape of the patient is fixed as a temporary shape using heat. Hence, when the patient places the aligner in their mouth, it fits perfectly, and the shape evolves from the temporary to the fixed version using oral temperature, ensuring a better fit and improved comfort. The main issue is that if the treatment has inaccuracies, the initial effect of the temporal shape is lost. Additionally, these polymers change their shape based on a combination of temperature and humidity in a quite narrow window and, thus, may not work for all cases [24]. Most SMPs are toxic, rather expensive, and difficult to manufacture at the industrial level, so it is rather difficult to implement SMPs for only a minor improvement that may be lost easily.

That is why the second approach, which is based on producing aligners with two or more shapes, is preferred. Once the aligner is handed to the patient and it has been worn for the prescribed period, it may be reshaped to the next step using heat and humidity [23,25]. Both approaches can be made using the same material, but finding a transparent material with similar mechanical features to conventional thermoplastics, biocompatibility, and shape memory effect is a challenge nowadays. For instance, there are already commercial solutions [26] that are either directly 3D printed or thermoformed, using step-shifting aligners where the use of only one aligner for two steps in the treatment is carried out by reforming it via immersion in hot water for a few minutes, although scarce information is available on the effect of using the aligner regarding its capacity to be reshaped or its properties and the forces exerted by this type of materials.

Regarding 3D resins, printing the aligners directly will bring many benefits due to a better fitting of the device to the dentition [27] and a faster, more economical, and more ecological production process since many of the steps comprising molds and material cutting would be eliminated, with material waste reduced [14,28].

## 3. General Requirements of Thermoplastics for Clear Aligners

The principal requirement for CAT is a transparent and biocompatible plastic able to sustain both conditions for at least 14 days in the oral environment. These conditions are held for almost all the polymers already listed, but secondary requirements may be more important nowadays.

The second requirement is good biocompatibility, or, in other words, low cytotoxicity. In this requirement, low leachate emissions and low water shrinkage [12,14] are needed.

Regarding the mechanical requirements, the material should have a low but sufficient elastic modulus—enough to perform dental movements effectively but not too high so as to cause pain or damage to soft tissues or to be uncomfortable. Some simulations have demonstrated that an elastic modulus of around 550–650 MPa is more than enough to perform most of the movements [29,30,31,32,33], but it is important to know that the pain and dental movement force thresholds are patient-dependent and may be very variable; thus, setting a definitive value for any mechanical property for all the treatments would be misleading [34,35,36].

## 4. Optical Properties

The main requirement for a material to be used in clear aligners is transparency and longevity throughout treatment. Thus, generally, fully amorphous materials are preferred over crystalline ones [10,12]. In these requirements, we can include the capacity to maintain transparency for the duration of the treatment and not be affected by common staining agents such as coffee, black tea, pop soda, red wine, or nicotine. In this case, color shifting after hours or days of exposure to the different agents against non-exposed samples or others exposed only to water is measured [28,37,38,39].

The most common method to measure the optical transparency in thin plastics is spectrophotometry, which measures the light intensity or the number of photons that reach the sensor and pass through the material to be characterized. Usually, it is measured against a reference, which could be air or a reference plastic sample. This method requires flat samples with enough area to obtain reliable results. Thus, devices are not usually measured in this manner; only flat sheets before being transformed are measured, although some studies have achieved otherwise by sectioning parts of the device and adding accessories to fix the position [28,38].

To measure the devices directly, usually, a dentistry colorimeter is used against non-exposed samples so as to only characterize the color shifting based on different color systems [37,40]. The main problem with using a dentistry colorimeter is that these devices work under reflection light configurations and are calibrated to measure the color difference of the teeth enamel; thus, the sensitivity may be poor when comparing two types of plastics. Therefore, measurements are compared against the signal of the bare teeth of a patient, which can be misleading since the enamel color may vary from patient to patient. Thus, the use of a reference is preferred.

A more complex system, comprising a flatbed scanner and a calibrated white background, has also been used to characterize color change directly on clear aligners after immersion for 14 days in different solutions [41].

The main conclusion of all studies is that the aging and wear of plastics, as well as the presence of saliva and other chemicals, ultimately decreases transmittance after 7 days [37,38,40,42], although more recent analyses report an improvement in these properties in recent years [28]. The most transparent options are PETG, TPU, PC, and multilayered materials; no significant differences were found between those materials in a naive state. In all cases, transparency decreases after the thermoforming process due to increased surface roughness [40,42].

**Figure 2:**
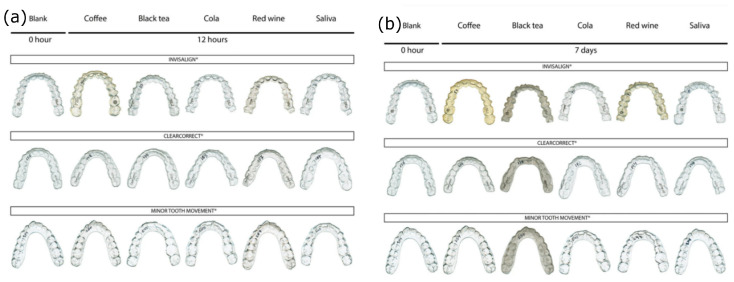
Aligners stained by various common staining drinks. [ClearCorrect (TPU), Invisalign (multilayer), and Minor Tooth Movement (PETG)]. (Reproduced or adapted from [41] with permission from publisher BMC, 2020). (**a**) Staining after 12 h of immersion; (**b**) staining after 7 days of immersion.

The main difference comes from the resistance to staining agents and the uptake of water. The most susceptible material seems to be TPU due to its major water sorption and porosity [41], and the most damaging substance is coffee as it can be observed in Figure 2 [37,38,40,41,42].

We must warn that the Invisalign material has a unique structure for a multilayer, having soft outer TPU layers and an inner polyester core; this is the reason why it is more prone to staining than other multilayers with the opposite structure as it can be observed in Figure 2, and this enforces, even more, the fact that TPU is the most susceptible material to coloring [38,43].

## 5. Aging and Chemical Resistance

The most common way of testing aging is to submerge the material to be tested in water or artificial saliva at 37 °C for a required time and test a mechanical property afterwards in comparison to a non-aged material, although other solutions have been proposed to simulate the action of conventional chemicals in food and beverages. Usually, the tests are performed on a non-thermoformed material [19,43,44,45] and even over a final aligner [43,46,47,48], but it is more common to test a simulated tooth to obtain a plain surface [45,49].

Higher water sorption is generally related to decay in all the mechanical properties, specifically in stress relaxation, with an increase in the stress relaxation and a decrease in the initial force; for instance, one of the reasons PC is no longer used as a CAT material is that it undergoes dramatic hydrolysis after long periods of immersion in common liquids, as water impoverishes its mechanical properties [10]. In contrast, soaking TPU in acid solutions decreases the relaxed stress and increases the elastic modulus of the material, supposedly due to a chemical reaction in the material [47]. Biofilm deposition may also affect the mechanical properties, increasing the fragility and hardness in almost all cases [46], whereas immersion in water and cleansing agents seem to make PP and PETG devices more plastic, but no high differences were found between aging in water, cleansing agents, and dry specimens in cracking fracture energy [50].

Some studies compare the properties of worn aligners after some time of usage. These studies show that the elastic modulus and the indentation modulus both decrease slightly after 7 days of use, but the values do not vary significantly after 14 days. The initial stress and the final stress in stress relaxation also decrease after usage but these values can be misleading because only one strain of 0.6% was utilized in Invisalign aligners [51]. The main conclusion is that Invisalign aligners lose elasticity after usage [51,52,53]; however, there were no similar studies for another kind of thermoplastic. In another similar study performed on 3D printed aligners using the resin TH-85, no significant change in properties was found between the used and non-used samples after 7 days of use [54]. In a deeper comparison, although Invisalign [52], PETG, [55], and TC-85 have similar values of elastic modulus by indentation, the stress relaxation in Invisalign was much lower (4% in non-used samples, and 10% after 1 week [52]) than in TC-85 (45% before and after use [54]). These findings suggest that although 3D resins may have similar elastic properties, they are easily deformable and, thus, not good for CAT.

Interestingly, blending and stacking layers seem to decrease the water uptake and, thus, are supposed to improve the aging of these devices. In a study, all the PETG/PC/TPU blends showed a lower water absorption as compared to commercial PETG products after 14 days of immersion at 37 °C [19], whereas, for multilayers, the results seem controversial. The only study addressing this matter consisted of a bilayer of PETG and TPU reinforced in some points by a rigid resin of unknown composition. The comparison with commercial materials shows that although the bilayer points absorb much more water than a monolayer, including a third layer decreases the water intake so as to lie in the middle of both. These results seem to confirm that TPU is one of the most hygroscopic polymers used in CAT [16].

Some plastic brands make use of a test known in the industry as the “Mustard test”, which consists of cutting a rectangular stripe of material and wrapping it around a cylinder to generate a known stress and strain level in the sample. Afterwards, the cylinder is submerged in a chemical, usually mustard. After a certain number of days, the sample is removed from the mustard bath and unwrapped over a plain surface, and the bending angle and coloration are measured after 1 h of recovery. Then, the sample is observed using optical microscopy, searching for microcracks, and finally, a mechanical test is performed on the sample [56]. The idea of this test is that a liquid seems more easily able to penetrate an amorphous polymer in areas of stress concentration; thus, these areas would be plasticized, decreasing the glass transition and the mechanical properties, thanks to increased local chain mobility, generating microcracks, which grow and coalesce into larger and fatal cracks. The main disadvantage of Mustard tests is that the material is never subjected to such high strains, nor is it fully immersed; thus, the values of the combined effects are overestimated. A very similar test was performed using pop soda. The study consisted of immersing thermoformed samples of different PETG brands in artificial saliva for 2 weeks at 37 °C and in pop soda for 10 min each day, measuring the flexural modulus using three-point bending (3PB). No significant difference was found between immersing and not immersing the sample in pop soda and between different brands of PETG [57].

## 6. Mechanical Properties

The devices should exert a slight force for around 22 h a day at 37 °C, immersed in a humid environment. Thus, it is important to characterize the elastic modulus, but it may be even more important to describe the elastic range of the plastic. Clear aligners need a reliable plastic range because the environment and the temperature will only decrease the elasticity of these materials [45,51,52,58,59,60].

**Figure 3:**
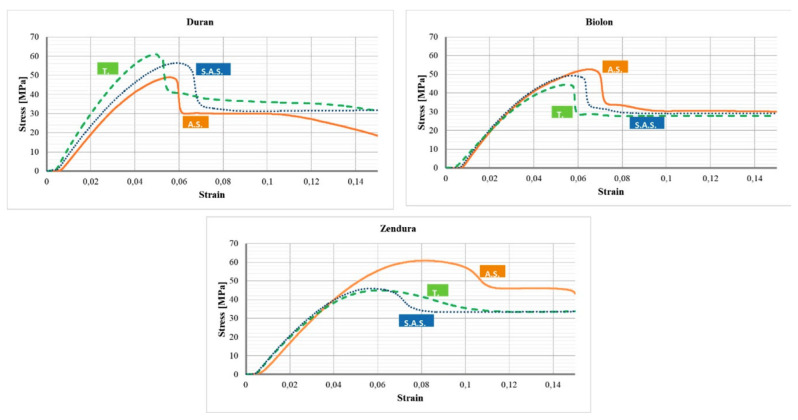
Stress–strain curves in PET (Biolon), PETG (Duran), and on hTPU (Zendura). T-Thermofomed materia, A.S—As served by the distributor, T—Thermoformed, S.A.S—Agend in artificial saliva(reproduced or adapted from [60] with permission from MDPI AG, 2020).

As we can observe in the Figure 3, the shapes of the PETg and hTPU stress–strain curves are fully different. Clear aligners are designed to act within the elastic range and near the yield point [61]. If the strain applied is lower than the yield point, the effective force is much lower than the planned one, and thus, the efficiency drops. However, if the strain is larger, the device is mechanically plasticized, deformed to the dentition, and ceases to enact force. In hTPU, the security range is broader than in PETG, as we can observe from the images, so the forces vary less. Moreover, we must take into account the fact that since the devices move the tooth but mold gradually to the dentition, the strains applied decrease gradually each time the device is removed and inserted again. In the case of PETG and PC, the patient would observe that the first cycles are painful, but the rest (since the force is much less) are more comfortable. In hTPU, since its elastic range is larger, the cycles suffer less variation in applied force, which is also lower, making the devices more comfortable. Thus, three factors are crucial: the yield stress, the yield strain, and the elastic modulus.

**Figure 4:**
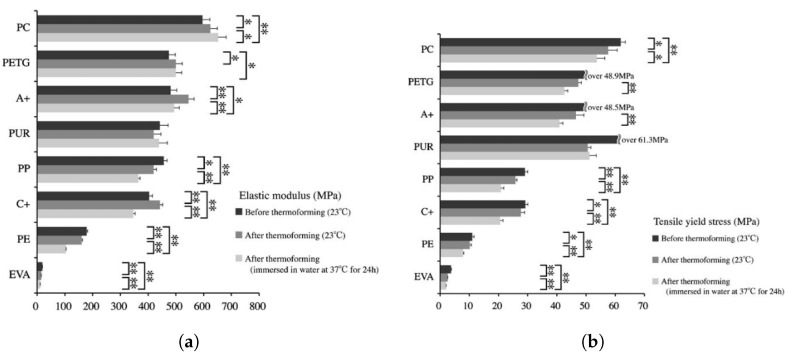
Elastic properties from tensile test in different clear aligner materials. [A+ (PETG) and C+ (PP)]. Statistically significant differences: * *p* < 0.05, ** *p* < 0.01 (reproduced or adapted from [45] with permission from Tylor & Francis, 2019). (**a**) Elastic modulus; (**b**) yield stress.

Regarding yield stress, PC has the highest value and the highest elastic modulus, but hTPU can reach comparable values in yield stress, having a lower elastic modulus. PETG, on the contrary, generally shows a higher modulus than hTPU and a lower yield point, as it can be observed in Figure 3 and Figure 4. [12,45,62]. PET and PC seem to have very similar values in elastic modulus and yield stress and strain, but the addition of ethylene glycol to PET (to make it amorphous, easy to work with, and more economical) seems to decrease the elastic modulus and the yield stress and increase the yield strain [63]. Adding PC to PETG seems to increase the yield point and the elastic range of PETG, creating a better material, but more research on that combination is required [20]. Another study also included hTPU in the blend, but the testing was carried out on tearing rather than tensile or 3PB, which makes it difficult to compare with other studies [19]. SMPs and 3D resins seem to have an elastic modulus similar to those of PETG and hTPU [64,65,66,67]. Table 1 resumes the main features of the most common thermoplastic used in clear aligners.

The most common manner of measuring the mechanical properties in plastics is the three-point bending test (3PB) due to its simplicity, sometimes enacted in a water bath at 37 °C, but most clear aligner plastics are rarely mechanically plasticized at the end of this method; thus, it is not as informative as the uniaxial tensile test because, in flexion, the materials rarely fail and deform permanently at rather high strains [38,57,67,80,81].

Finally, the last method used to measure the mechanical properties of these devices is indentation directly in used aligners [52,53,55]. This method allows punctual determination of the viscoelastic parameters but relies on fitting the indentation curves, and the values may be affected by the viscoelasticity of the material itself [82]. In this way, it has been reported that Invisalign appliances decrease in elastic modulus after only 7 days of use, but the drop is stable after 14 days, while the relaxation modulus suffers an inverse relation, which represents another way to ascertain that the material becomes more plastic after usage [52]. This fact is supported by other studies that have found a drop in the elastic modulus after aging using temperature and storage in certain media such as saliva or water as can be seen in Figure 3 and Figure 4 [38,60].

The same software used in nano-indentation usually estimates the hardness of the material. However, it can be measured using other methods, which can usually be applied only to flat samples of a specified thickness (Vickers, Knoop, or shore hardness). PETG, PC, and hTPU monolayers are reported to have very similar values in hardness, whereas multilayers are generally slightly softer; finally, PP displays the lowest hardness [55]. In theory, the hardness should decrease with water absorption in simulated aging but increase due to cold working and biofilm deposition on the surfaces after usage of the aligners. However, it is reported that water sorption is more powerful than the two effects combined [52,53]. It is reported that the oral pH may vary from 5 to 9, being generally acid [83], but scarce information is available on the effect of acidity on the mechanical performance of these materials, even when one of the most used (hTPU) may undergo chemical alterations in low pH values [47].

### 6.1. Orthodontic Force and Strain Measurements

Computing the actual force acting during treatment may be difficult, so the most common ways to address this issue use the simulation of treatment with finite elements or measuring the force over a 3D printed dental model from an oral scan.

**Figure 5:**
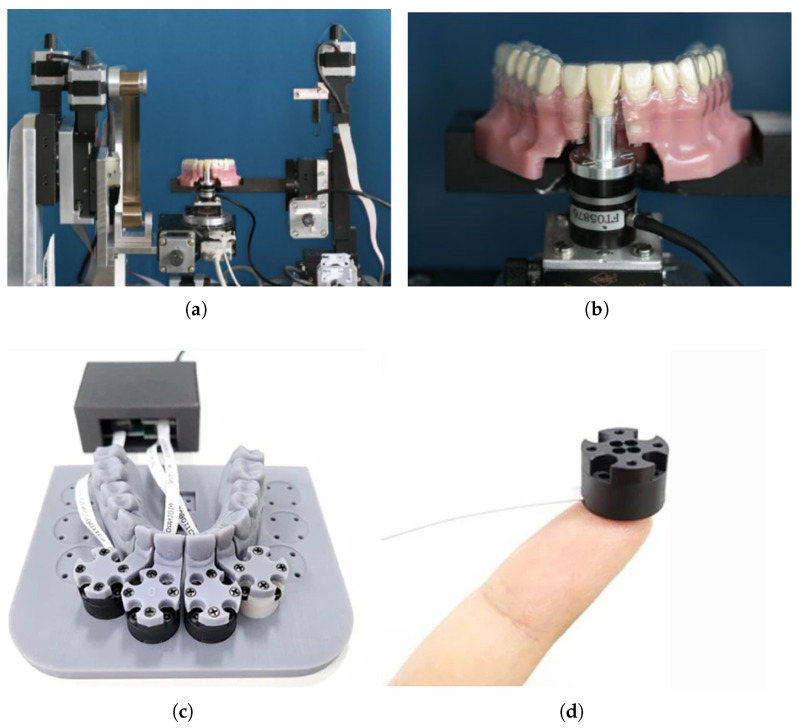
Different OMSS equipment. (**a**) Reometer-based OMSS (reproduced or adapted from [89] with permission from Springer Nature, 2015); (**b**) dentition setup with an aligner in OMSS (reproduced or adapted from [89] with permission from Springer Nature, 2015); (**c**) six axes force and moment sensor OMSS (reproduced or adapted from [90] with permission from Springer, 2023); (**d**) six axes force and moment sensor (reproduced or adapted from [90] with permission from Springer, 2023).

To measure the force that can be produced in a treatment situation, directly building what is called an orthodontic measurement and simulation system (OMSS) is usually needed. These machines typically feature a dentition model with movable teeth and a system to measure forces and torques in three dimensions. What is typically carried out is the selection of a case study and positioning the OMSS accordingly. Finally, the aligner is placed on the dental model, and the force is measured. Examples of OMSS set up can be found in Figure 5. The main drawback of this technique is that the OMSS cannot be immersed; only temperature can be applied, so the aging can only be applied before the testing and not in situ. Only static forces were found in the literature, and the effect of time or of moving the teeth simulating real movement is not reported, although, in principle, the system can measure it [11,17,18,84,85,86,87,88,89].

Another simpler solution has already been envisioned by filling the tooth hole with a wax that simulates the soft tissues and through which the tooth is nailed. This method requires placing the tooth and freezing the wax before introducing the device into the OMSS at 37 °C and setting it to perform a constant force. At the end of the simulated treatment, the displacement achieved can be computed by comparing an initial scanning of the mold with the final one. Using this method, it has been proven that SMP reforming each week using one aligner per two phases achieves larger or at least comparable displacements than conventional thermoplastics, but these results are dependent on having similar properties to the soft tissues or at least being able to produce movements at the same pace [23].

A similar method to OMSS is the digital image correlation (DIC) method. This is based on two digital cameras placed at certain angles, focusing over a clear aligner that is tinted with a black-and-white spot pattern, placed over a dental model. The strain is measured from the separation of the spots based on a calibration, and if the elastic properties are known, the force and stress may be computed [18,91,92,93]. Conventionally, the values of stress, strain, and forces are given as resistance against plastic deformation. The problem with this method is the complexity of the analysis of the images and the painting of the aligner reliably. No aging or temperature may be used in this method to avoid affecting the paint, but it is reported that it is possible to apply this in vivo by pasting a spotted pattern paper directly to the dentition of the patient [93].

**Figure 6:**
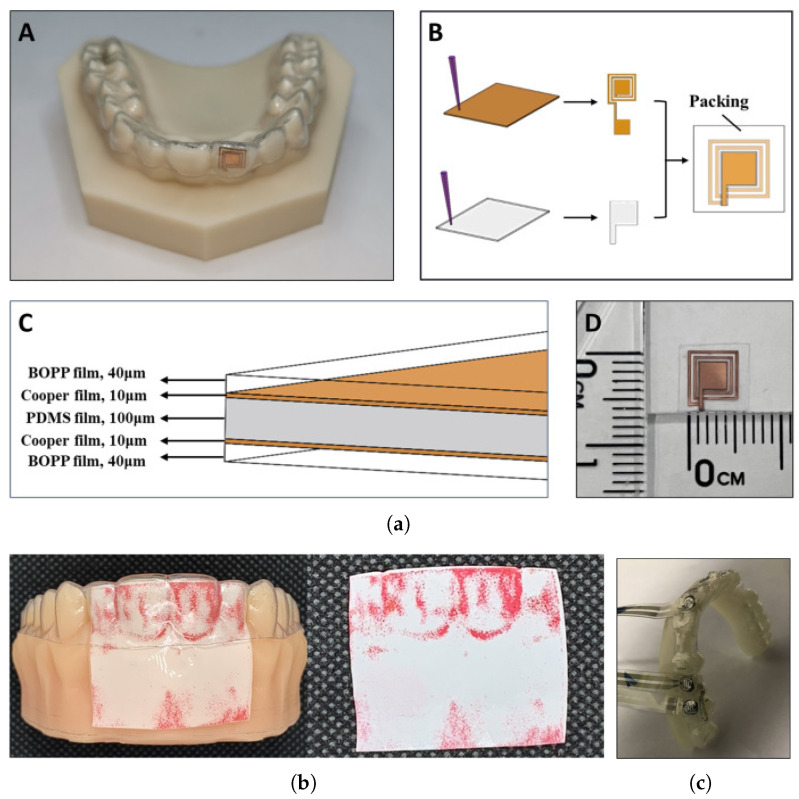
Methods to measure the force on aligners. (**a**) Electromagnetic force sensor (**A**) Sensor attached to a clear aligner measuring the force, (**B**) Sensor builing diagram, (**C**) Sensor structure (**D**) Final sensor just after fabrication (reproduced or adapted from [106] with permission from Elsevier, 2024); (**b**) thin force-sensitive films (reproduced or adapted from [94] with permission from Elsevier, 2022); (**c**) wired force sensors attached to an aligner (reproduced or adapted from [100] with permission from Elsevier, 2019).

Another way to perform this is to use a force-sensitive paper or film between the aligner and the dental model to measure the orthodontic forces, what can be seen in Figure 6. This paper changes its color depending on the magnitude of the force, and thus, the analysis can be misleading. Moreover, placing a layer between the aligner and the model may overestimate the force values [11,94,95]. A study comparing forces obtained from the OMSS setup and the thin-film techniques demonstrated that OMSS measures forces that are nearer to the biological range than thin films [96]. The same study also shows that with both techniques, the decay in force after aging in multilayers was much less than in monolayers of hTPU and PETG. Both methods reported similar values in force difference. Regarding 3D printed clear aligners, the resins have been proven to be much softer than the thermoplastic in use, and thus, the forces are much lower, meaning it is still controversial if any effect would be achieved using such low forces [66,78,90,97,98].

Similarly, attaching or bonding a capacitive force sensor to the aligner directly and measuring the force against the plastic model has been used successfully, reporting similar values to OMSS. A capacitive sensor can be seen in Figure 6 attached to a clear aligner to measure orhtodontic force. This technique also adds an extra layer to the aligner, which results in an overestimated force value, and the wiring can affect the fit of the aligner and the model [99,100,101,102,103]. Recently, some researchers developed a piezoelectric sensor with minimal thickness, which can be seen in Figure 6 and the ability to measure wirelessly the force based on an electromagnetic coupling effect. Unfortunately, this study was only a demonstration of the working principle, and only in vivo immediate force was recorded in a specific situation [104].

All the methods have been used to demonstrate that the force generated by multilayered aligners is more gentle than in monolayers, being 2–3 times lower than in PETG and 1.5–2 times lower than in hTPU in models, and 3D resins enact almost no force compared with thermoformed aligners [11,17,18,78].

OMSS has been used to address the problem of activation as can be seen in Figure 9. Activation is the misfit between the dentition and the aligner, defined in mm. Conventionally, all the clear aligner systems are designed under an activation of 0.25 mm. The initial forces increase with the activation, but after some days, the forces decay to the same threshold value. Thus, higher activation forces would only mean using forces over recommended values, which will quickly decrease [104,105].

Relating the damage caused to the soft tissues with the applied force can be a very useful tool in biology to understand the tooth movement process. It is possible to measure the damage by measuring the concentration of the lactate dehydrogenase (LDH) enzyme in the saliva. This enzyme is restricted to the intracellular environment; as cells receive damage, the LDH levels increase in the extracellular environment due to an increased permeability of the cellular membrane. It has been possible to measure the damage caused by metallic appliances using different forces from 40 mgf to 80 mgf for 21 days in real patients [107] and only 125 mgf in another study [108], but no study was found applying this to clear aligners. The tests consisted of taking salivary samples of the patients and measuring the LDH concentration level using a spectrophotometer, comparing the data against the pretreatment values or the saliva of a non-stressed area of the same patient.

### 6.2. Stress Relaxation and Creep

**Figure 7:**
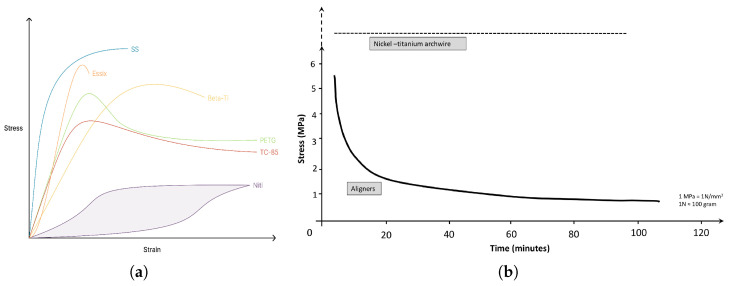
Mechanical properties and stress relaxation curves of different materials used in orthodontics. (**a**) Stress–strain curves of different materials used in orthodontics. (reproduced or adapted from [14] with permission from KeAi, 2023); (**b**) stress relaxation in metals against plastic aligners. (reproduced or adapted from [109] with permission from Elsevier, 2022).

The main property used to address instructed professionals in CAT is stress relaxation. Clear aligners are designed to mimic fully elastic metals, which are deformed elastically to a certain percentage and can keep the force stable for a long time with minimal plastic deformation. In plastics, this does not happen because plastics are viscoelastic materials and thus suffer a viscous force drop until reaching a stable force value that is used to settle the position, what can be seen in Figure 7. The elastic initial force and the shape recovery when not worn depend on the elastic features of the material, but the force drop over time and the fitting to the dentition when introduced are dependent on the viscosity [47,58].

The viscosity features of the aligner can be seen as a creep. A small creep may be beneficial to dissipate excessive forces over the periodontal ligament and to ease the fitting and removal of the aligner, but excessive creep will compromise the effectiveness of the aligner [47,58].

Creep is the setting of a defined stress level for some time and monitoring the deformation. The main issue of creep is that it does not represent the reality of a clear aligner because the strains in an aligner are defined by the velocity of the tooth movement and the deformation history of the material. Another drawback of creep testing is that forces in creep testing are too high from the real ones, which also gives strain values far from the ones in clear aligners [47,58]. Thus, it is preferable to set a strain level for some time and follow the forces and stress, which is called a stress relaxation test. The strain level to be set is also controversial in stress relaxation, and conventionally, a value of 0.6% [47,51] is used, but it has been demonstrated that in clear aligners, strain spans from 0.01% to 1.8% [92]. Another way to perform this would be to take a strain in the material in the linear elastic region far from the yielding point to assure elasticity [44,48,49], but this approach uses stresses or forces far from the ones in treatment.

The way to measure stress relaxation or creep is by using a conventional universal testing machine in tensile [44,47,49,110] or 3PB configurations [48], although others have used DMTA equipment to obtain more reliable results since it can measure lower forces [47,58,66,111], as well as perform cyclic testing resembling aligner fitting and removal [58,64]. From the cyclic testing, it is clear that both in creep and stress relaxation, the material is more viscous after usage [58] and after simulated aging [64]. The study compares PETG and TC-85, a 3D printing resin for clear aligners, at 37 °C and 80 °C. In both materials at 37 °C, the initial force of the stress relaxation stages decreases until reaching a plateau value after five cycles; the same effect is observed in the strain at the end of the recovery stage, which increases until reaching a constant value. These effects are more noticeable in the TC-85 resin due to the higher deformability of this material, and the force retention in the stress relaxation stage is extremely poor, being only 10% against the 80% in PETG for the first cycle of both materials. At 80 °C, the shape recovery of the 3D resin can be observed as a negative strain in the recovery part that increases toward zero with the cycles.

A very similar study was carried out on SMPs, based on deforming the material at a temperature over its Tg to settle a temporary shape and cooling down the material while keeping the force constant [22]. A series of thermal cycling processes above the glass transition temperature (Tg) were conducted, during which the material consistently reverted to its pre-programmed deformed configuration, accompanied by an increase in recovery stress. Nevertheless, the findings indicate that the shape memory behavior deteriorates after a limited number of cycles. The stress relaxation profile reveals a partial recovery component, and despite the relatively low number of cycles (10), a progressive accumulation of residual strain was observed with each cycle, an effect also reported in materials such as PETG and TC-85.

The stress drop in hTPU materials, in general, is more gentle than in PETG monolayers, but the initial stress or forces are usually too high in both materials, although higher in hTPU than in PETG. The worst option is PP, which can be easily deformed and suffers the highest force drop compared with PETG and hTPU [110], although this study was performed setting a continuous force level of 140 to 108 MPa in immersion. Multilayered materials decrease the initial force and maintain a gentle force drop thanks to the use of a soft layer [47,48,49], whereas the blending of PETG with PC seems to only decrease the force drop [20]. Aging and use seem to decrease the initial and final forces and decrease the force drop. It is interesting to note that hTPU may undergo some chemical changes under acidic environments that decrease the force drop compared with artificial saliva aging [47]. Stress relaxation for long times (200 h) in PETG materials has demonstrated that the force keeps decreasing for long times, but this material suffers sudden force drops followed by a slight increase in the force, which seems to be related to the development of slip bands in the material [112].

**Figure 8:**
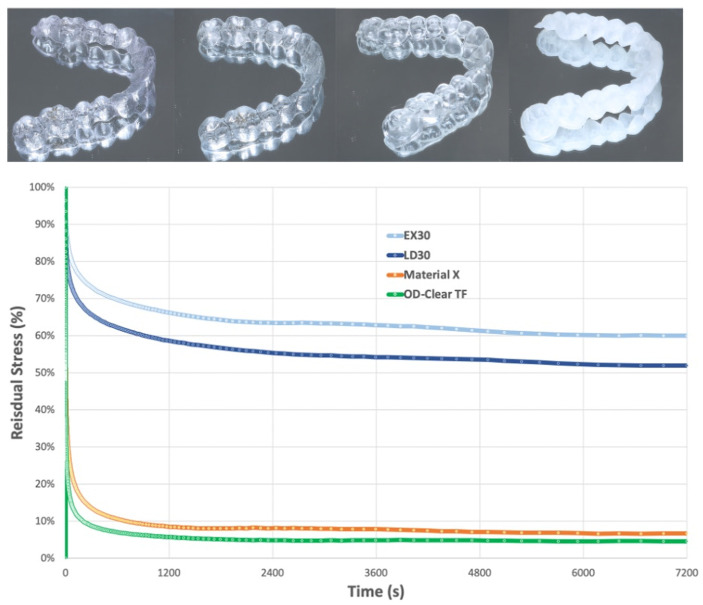
(**Upper**) Clear aligners; from left to right: EX30 (TPU), LD30 (Multilayer), Envisiontec material X (3D resin), and OD-Clear TF (3D resin). (**Lower**) Stress relaxation of thermoformed and 3D printed aligners (reproduced or adapted from [66] with permission from Elsevier, 2023).

One of the most problematic issues in direct 3D printed aligners is stress relaxation as seen in Figure 8. Although resins currently approved for this purpose can perform lower forces, they cannot sustain as much force as thermoplastics, mainly due to fast softening of the resins [66,97].

Creep and stress relaxation have been measured, too, using nano-indentation over the surface of aligners, only in the Invisalign multilayered material [52,53]. The creep index, seen as the increase in indentation depth between the initial creep time and the final, seems to increase in used samples [53], and the relaxation index, as the force decreases between the final and the initial stress relaxation tests, seems to increase [52]. Another fact in favor of this hypothesis is that the forces and elastic modulus are lower in the used samples.

**Figure 9:**
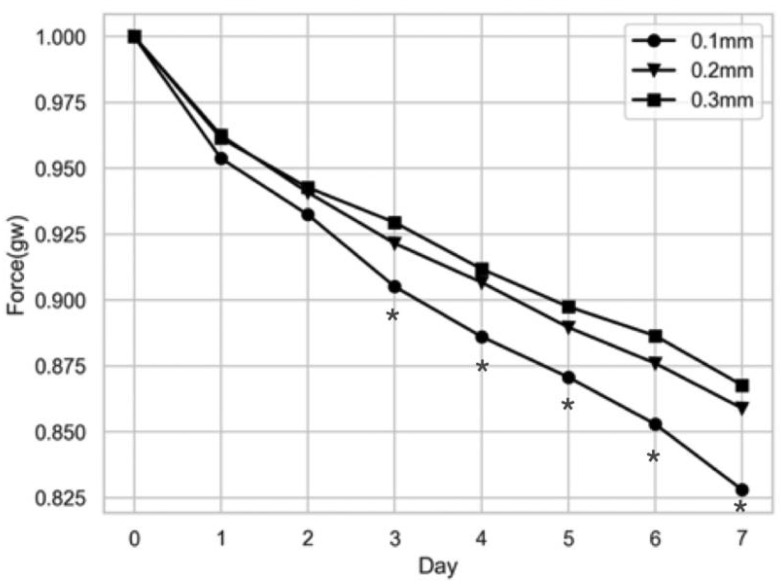
Measured stress relaxation curves with OMSS in a PETG aligner with different activation distances (reproduced or adapted from [105] with permission from Elsevier, 2023).

The force sensors previously mentioned have been used to measure force degradation over time, as seen in Figure 9, more realistically. Still, scarce information is available in the literature regarding this [87,96,105], although it seems to confirm that the material is more viscous when used and that the force drops following a straight line, as can be seen in the Figure 9 [105].

## 7. Thermal Properties

The glass transition in polymers is a measure of the ability of the polymer chains to slip and move and, thus, dissipate energy-producing deformation. We must take into account that clear aligners are worn for 22 h for 15 days at 37 °C in a humid environment, which promotes polymeric chain movements, meaning permanent deformation. Thus, a high glass transition means less permanent deformation and, thus, more stable treatments; however, it also serves to set the thermoforming parameters. Although most of the CAT plastics are thermoformed using the same procedure, with slight changes in heating time, it is reported that TPU is more accurate at reproducing the shape of the molds than other materials, although its Tg is slightly higher than PETG [14].

The usual method to measure the Tg uses the heating and cooling profiles of DSC, usually from 20 or room temperature to 300 °C. Another option would be to perform DMTA tests, which consist of exerting a cyclic deformation over a material either in tensile or 3PB configuration while the temperature is increased continuously. From these measurements, the relaxation between the imposed signal, the strain, and the response of the material, the stress, is obtained as the complex modulus and decomposed into its real (storage modulus) and imaginary (loss modulus) states. The storage modulus can be seen as the elastic energy stored in the system, and the loss modulus is the energy lost in viscous deformation. Conventional thermoplastics are usually tested from 0 or 20 °C to 200 °C with a deformation frequency of 1 Hz, and the Tg is taken as the maximal point of the loss modulus profile. However, not only is the Tg important from these experiments, but also the values of both moduli at 37 °C. TPU shows a larger loss modulus value than PETG, which can be related to a better fitting of the appliance and, thus, better efficiency [113]. Three-dimensional resins were found to have much higher loss moduli than those of PETG and TPU; thus, it may be hypothesized that 3D resin aligners will deform quickly to the dentition shape and cease to make any force [64]. A similar situation happens with PVC Polyvinyl chloride, the loss modulus of which at low temperatures is much superior to those of PETG or TPU [114], meaning the usage of PVC aligners is restricted to 14 h per day to avoid mechanical plastification [77].

Conventional materials such as PETG and TPU show glass transitions fairly near the oral temperature (75–80 °C for PETG [37,47,115] and 80–95 °C for TPU [37,47]), and thus new materials with higher glass transitions have appeared [37,116]. This factor seems to be of high importance in 3D printing since the only material already approved for direct printing aligners seems to have a Tg at 37 °C, which is reported to be the cause of extremely low efficiency and low forces compared with thermoformed aligners [64,78,90,98].

The thermoforming and the presence of water decrease the Tg in all the polymers tested, but the thermal properties do not seem to change after aging in thermoformed PETG samples [115]. In the case of TPU, this material has amorphous and crystalline phases. It is reported that the melting enthalpy of TPU after thermoforming decreases, and the glass transition shifts towards lower temperatures, meaning an increase in the amorphous phase [51]. The literature lacks a study addressing aligners’ thermal properties after usage, but minor changes should happen, most of them due to water sorption, causing a drop in the glass transition.

## 8. Biocompatibility

### 8.1. In Vitro Testing

Mainly, all the studies to ascertain the biocompatibility of clear aligners are based on measuring the metabolic activity of oral cells when exposed for 15 days to an aligner and measuring the activity of some enzymes via colorimetric methods [39,68,117]. Most materials have low or null cytotoxicity using this method, and the only ones with health risks are novel materials used for direct 3D printing [79] or SMPs [118] only because they are yet to be tested deeply, although the current findings suggest similar or even better scores in cell viability testings between thermoformed, SMP, and 3D printed aligners [119,120].

Other concerns have arisen recently regarding microplastics: [121,122] leaching in terms of saliva or water [110,123,124,125], histologic reactions [126], estrogenic effects [117], and the emissions of endocrine disruptors like bisphenols [127,128] and phthalates [129,130,131]. For example, one of the most limiting reasons regarding the use of PC in medicine is that PC is produced from bisphenols, but bisphenols are only a risk for the human body when they are unreacted or used as additives for ease of production [72,128]. Nowadays, bisphenols in commercially available PC are fully reacted and cannot leach into the body tissues, so they can be labeled as BPA-free material. Even having this indicator is not a reason to be fully secure since many plastic manufacturers outside the EU and USA use this label even knowing their product has BPA levels over the permitted threshold [128].

BPA can also be found in some TPU formulations like the one in Invisalign [123]. Previous studies have reported that BPA leachates are in low amounts, far below the health risk threshold, or even undetectable in clear aligner materials. In general, TPU and PETG used for clear aligners release almost undetectable amounts of BPA, although in the Invisalign material, BPA was detected in higher amounts. Regarding other bisphenols, TPU and multilayers, in general, may release BPF, whereas this chemical was found in negligible amounts in PETG. Finally, BPS leachates were found to be negligible in all the materials. Although these results were on leachates from aligners after 8 weeks of exposure to a saline environment, the amount after 2 weeks of oral exposure should be minimal for all bisphenols [123,124,125]. That is why no estrogenic effect was observed, at least in Invisalign appliances [117], after 2 weeks. These studies used liquid chromatography, first introducing a piece of aligner in the solution to be tested for a specified time, and then the liquid must go through an extraction process to remove salt from the solution to minimize the suppression of the target ion signal strength [123]. However, others have used FTIR-ATR to detect chemicals in the solutions, albeit with less success because this method is unable to detect chemicals in ppm quantities [110].

New BPA-free plastics have appeared on the market to replace PC and other plastics. Although these plastics are not BPA-derived, they do not lack risk; in fact, all of the BPA-free polyesters (including PCTG) have detectable estrogenic effects in vitro, unlike PETG, which was found to have no detectable estrogenic effect. Unfortunately, TPU was not present in this study. In this case, the approach was different; instead of detecting any chemical with a reported estrogenic effect, the material may leachate; the test used two special cancerous cell lines. The first cell line type proliferates only under estrogenic ambiance, and the other cell type responds to the estrogenic ambiance with an activation of the luciferase firefly enzyme. The first test quantifies the amount of estrogenic activity via the cell population, whereas the second one utilizes a luminescence method to measure the estrogenic activity in terms of luciferase produced [72]. A similar test was utilized to measure the estrogenic and androgenic activity in PCTG monomers, concluding that the monomers, at least, leach no estrogenic chemicals, but the methodology to obtain the leachates was much more aggressive in [132] than in [72], and thus the results are not comparable. Nevertheless, there is no list of all the estrogenic-active substances that may be released from plastics, their effect on fetuses, children, and adults, and the health risk thresholds. Thus, it is not fully clear that PCTG and other BPA-free products are non-estrogenic, but the same is true for other types of plastics, such as TPU.

In the case of histological reactions derived from clear aligners, no report was found on PETg or in most multilayered materials, despite the increase in reported cases of contact allergy to PEG [133,134,135,136] and the health concerns regarding phthalate emissions. It is reported that Invisalign material may cause reactions due to the isocyanate present in its TPU formulation [46,137,138,139]. No cases of allergy or response were found in other multilayer brands having a copolyester as the outer layer.

Biofilm formation is also an interesting feature to have a look at because bacteria and other microorganisms attach to the surface of the aligner and may promote different illnesses such as cavities or periodontitis but may also attack the plastic by leaving calcium residues that may end up decreasing transparency and creating microcracks. The most common methods to test biofilm formation are scanning the aligners’ surfaces in SEM [140], bioluminescence methods [141], and counting the colony-forming units (CFU) using the bare eye [142]. The results are scarce but show no difference in biofilm formation and growth between materials and that this cannot be avoided; it is only alleviated by clearing the aligners when removed, preferably with commercial sanitizing tablets designed for this purpose [140,143].

To avoid biofilm formation and address the demineralization problems, covering aligners with gold nanoparticles has been proven to be effective [144], whereas the use of hydroxyapatite on functionalized 3D printed clear aligners has resulted in a slight improvement in the mineralization of the teeth, a decrease in mechanical properties, and an increase in the cytotoxicity of the materials is reported [145].

### 8.2. In Vivo Testing

The cellular response to a material may be very different from that obtained in the laboratory; thus, in vitro testing in animals is needed, especially for responses such as irritation. Afterward, clinical trials are performed on selected patients under informed consent.

For instance, it is said that chewing, speaking, and removing the aligners may lead to the irritation of the oral epithelial mucosa. In a recent study, it has been shown that the oral irritation of Invisalign and gold nanoparticle-coated Invisalign aligners is minimal, but in the conventional aligner, a slight erythema was observed [144]. The erythema is associated with the presence of certain types of bacteria and biofilms on the surface of the material. For instance, it was observed that pathogens and plaque scores are higher in metal fixed appliances than in clear aligners due to impoverished oral cleanliness, and irritation and erythema cases were more common [146].

Another interesting point is the feeling or opinion of the patient because it requires the complete cooperation of the patient for its success. For instance, it is said that TPU is more comfortable, especially in the first instances of treatment, and for the most sensitive patients, EVA clear aligners are even more comfortable than TPU ones [71,147], but we have found no clinical study supporting this evidence. It has been found that in rotation movements, PETG aligners tend to cause large root movements, whereas TPU tends to cause mainly crown movements with almost no root movement [148]. This finding implies that PETG aligners are capable of generating greater orthodontic forces compared to TPU aligners, which may constitute valuable information for clinicians when selecting materials to facilitate specific tooth movements, such as root displacement. In a separate study, the same authors investigated the interaction between bonded attachments and aligners fabricated from PETG and TPU. The results clearly demonstrated that PETG aligners induced significantly higher wear on the bonded attachments than those made from TPU [149]; thus, in a tooth with no attachment, the wearing would cause a reduction in the enamel, which can be accelerated in acidic environments and when extensively using the aligners without cleaning [150].

## 9. Environmental Effects and Recyclability of Clear Aligners

The recyclability of these appliances is a controversial topic that is starting to be studied. In principle, since clear aligners are cataloged as medical devices, they cannot be recycled due to general health risks and, thus, must be burned in specialized facilities, assuring that all the transportation is carried out under sterile conditions, although frameworks for the effective recycling and sorting of this type of material do exist [151]. Although most clinics have facilities to dispose of used aligners in clean conditions, in actual fact, most of these devices are wasted by the patients in their own houses as general house waste, posing an infection risk.

Another point that hinders recyclability is the secrecy of the composition of these plastics, but not many studies have addressed the issue of the contamination and carbon footprint of CAT or the reutilization of the aligners for other purposes [39,152,153,154].

Align Technology and other brands of the sector have recently started to recycle their aligners, which are sent to a third company that, after proper sterilization, remelts the plastics and utilizes them as hard plastic for recycled products [155], but the use as raw material for the same purpose is seen as undesirable due to the loss in properties [156].

Some experts have warned about this point recently and on the need for a clear way to dispose of the clear aligners [156,157,158,159]. In the meantime, some biodegradable products have also appeared, although scarce information regarding their real biodegradability and efficiency is already available for their usage as clear aligner products [157,160]. Moreover, green polymers from plant-based sources have appeared in the market. For instance, the substitution of terephthalic acid in PETG by furan dicarboxylic acid, giving polyethylene furanoate (PEF), is known to significantly decrease greenhouse gas emissions. PEF shows similar properties to PET, being mostly amorphous and more rigid [161,162], but no information regarding its biocompatibility and toxicity was found; however, very similar properties show that PCTG can also be fully obtained from plant-based sources [73]. Although most of the polymers used nowadays in CAT can be manufactured fully or at least partially from bio-based sources [163], controversy still arises regarding the use of food feedstock to produce plastic materials instead of their primary means.

In order to get rid of common polymer waste, a new method based on the enzymatic degradation of the polymer by suitable biological agents has been proposed. For instance, PET and PETG may be decomposed by bacteria or fungi such as *Ideonella sakaiensis*, *Streptomyces* sp., and *Phanerochaete*, *Chyrsosporium*, while certain types of polyurethanes can be degraded by *Pestalotiopsis microspora*, *Curvularia senegalensis*, or *Fusarium solani*, aided by the enzyme Serine hydrolase [164]. The main problems of this approach are having homogeneous waste from the same product and the economic availability of this process.

However, there is a major concern about the plastics used for clear aligners and their effect on the environment; nobody has actually asserted the life-cycle of CAT, from the plastic production and waste to the end of life of clear aligners, including secondary materials and issues, such as the disposal of the mold used for thermoforming, the packaging, and how the aligners are sent to the patient. For this, we can suppose that there is a huge difference between big companies that send the finished product globally and local vendors, from which the transport effects are almost negligible.

CAT may have a greater environmental effect than just the waste of a few grams of plastic from used aligners since all the steps of the process, from the polymerization of the plastic to final waste, must be included; additionally, secondary operations such as the molds used for thermoforming, the substances used to clean the molds from monomers and sterilize the final aligner, the packaging used to ship the aligners, and transport should be considered. We have to take into account, for example, the many materials used for producing the aligners and the fact that they are considered medical waste and, thus, are non-recyclable. Thus, an efficient LCA of clear aligners would include the following:

- Polymer manufacturing and processing to either flat sheets for thermoforming or resin for 3D printing;
- The aligner manufacturing process, including the energy consumption of the machinery used for thermoforming, 3D printer, and cleaning;
- Secondary materials, such as the 3D printed molds in the case of thermoformed aligners, manufacturing errors and wastes, cleaning agents, packaging, etc.;
- Shipment;
- The final disposal of the aligner.

However, 3D printed aligners and SMPs have risen as an alternative to reduce the use of thermoforming molds and decrease material waste in the process. This approach can also reduce the carbon footprint, but the resins available for direct printing and the SMP aligners are far more efficient, comfortable, and biocompatible than thermoformed aligners [11,14,22,28,64,66,121].

## 10. Perspectives and Research Directions of Clear Aligner Thermoplastics

It seems that most of the efforts are focused on directly 3D printing the aligners due to the clear advantage this would have on better fitting the appliances to the dentition [27] and also due to faster and more economical processes [14,28]. One of the main problems in 3D resins is the unknown cytotoxic effects. The scientific information regarding the biological security of these materials is still scarce, and doctors tend to consider this type of resin toxic. Thus, currently, only a few resin types have received FDA approval, and most of them are for mouth-guards or splints rather than for clear aligners.

However, 3D printing may not imply an increase in the efficiency of the process, which is the second point mostly researched by developing new thermoplastics with enhanced stress relaxation properties or by using aids.

One of the most promising materials is the SMPs, which are starting to be available commercially, but little information is available regarding their efficiency in the laboratory and in use and cytotoxicity.

Two factors that are currently the target of many specialists are the duration of the prescription and the use of soft and hard plastics for different types of movements. Mostly, all prescriptions are set to change the aligner every 14 days, using the same material all the time. Still, some studies have demonstrated that conventional thermoplastics may change aligners every 10 or 7 days with the same efficiency, but it seems that the decision depends on the easiness of the treatment and the patient response, with no clear guideline in this issue [165,166], as it happens with the use of two types of plastics for the different stages of the treatment. The prescription times can be further shortened using different types of aiders that are already under development, like optical attachments that accelerate the tooth movement while the patient is sleeping [167], vibrational tools that assure increased efficiencies and predictabilities using only 5 min of stimulation per day of treatment [168,169,170], or piezoelectric aiders that decrease the pain perceived for the patient and makes possible to also use even lower forces to move teeth thanks to faster bone regeneration [171,172]. In essence, vibrational and piezoelectric aids have resulted in increased predictabilities, efficiencies, and better pain perception, which would end up in patients being less prone to abandon treatments. Optical aids, on the other hand, only managed to use fewer aligners per patient, but no difference in pain perception, effectiveness, and predictability.

Most professionals rely on only one plastic for realizing all the movements, but specialized professionals and top brands use two types of plastics depending on the deformation level. In essence, if the teeth are perfectly aligned and only the expansion or retraction of the dental arc must be carried out, it is preferred to use a rigid plastic because the strains are small, and a soft plastic would not withstand enough force. In contrast, if the teeth are crowded, the deformations increase, and the rigid plastic generates pain at first and, after a few hours, loses efficiency, so a softer plastic able to deliver gentle forces for longer times is preferred. Thus, it is possible to align all the teeth first and then carry out the expansions or contractions or the opposite, but there are no clear guidelines on when to change from one stage to the other or how many rigid and soft aligners a patient would need.

Finally, we would like to mention the lack of studies linking the force level applied and the damage created to the soft tissues. This can be a promising tool in dentistry, not only to better understand the mechanobiology of tooth movement but also for patients since it may be possible to “personalize” the force for each patient. There are methods to tailor the mechanical capabilities of clear aligner materials and ways to measure how much force the patient can bear and how much damage the soft tissues receive, but there is no bridge in between. For instance, there are already LDH-sensitive papers [173] and sensors [174] that would make damage measuring fast and easy for an instructed doctor. Using that data, then, would make it possible to tailor the force for the patient in a second visit, introducing the data via software that takes into account the specific viscoelastic features of the aligner materials and the movements needed for each treatment.

We have summarized the main gaps of knowledge in CAT:

- The use of different plastics for each stage of treatment or for each type of movement.
- Implementing the material properties in the tooth movement software.
- The treatment efficiency and biocompatibility of 3D printed and SMP clear aligners.
- Prescription duration and activation distance.
- The use of aiders to speed up the treatment.
- In vivo force and strain measurements, including a comparison between fixed appliances and different clear aligner materials utilizing the LDH enzyme method like the ones reported in [107,108].
- Life-cycle assessment of clear aligners and their possible recyclability, as claimed in [153,154,156,158,159].
- Extend the literature on the biocompatibility effects of 3D resins and SMP, as well as on their effects on treatment and long-term exposure to this type of material, including variables such as the curing time and cleaning process in the case of 3D printed aligners and the release of noxious chemicals (BPA, BPF, BPS, phthalates, …).
- Extend the literature on estrogenic-active substances that may be released from medical-grade plastics, 3D resins, and SMPs, as claimed in [72]. This may include the following:
  – Standardized methods to measure the estrogenic activity of a chemical that leaches from a plastic.
  – The short and long-term effects on the human body of the estrogenic active chemical and the body uptake of the substance, all for different life stages, focusing on fetuses and children.
  – Define, if possible, health risk thresholds for that chemical.

## Figures and Tables

**Table 1:** Summarized table of general properties of materials used for clear aligner manufacturing. The biocompatibility grade refers to the percentage of cell viability in in vitro testing, with the following scale: 1 (0–30%), 2 (30–50%), 3 (50–70%), 4 (70–90%), and 5 (90–99%).

Material	E (MPa)	Tg (°C)	Tm (°C)	Transparency	Aging	Chemical Resistance	Biocompatibility (Grade)	References
PETG	2000–2200	80	-	Excellent	Good	Excellent	Excellent (4–5)	[14,39,46,60,68]
PET	2000–3100	80	260	Good	Good	Excellent	Excellent (4–5)	[60,63,68,69,70]
hTPU	2200–2500	90–120	150–220	Excellent	Excellent	Good	Good (4)	[47,60,62,68]
PC	2000–2500	145	297	Excellent	Poor	Good	Poor (3–4)	[12,20,62,71]
PCTG	1300–2300	100–120	-	Excellent	Excellent	Excellent	Excellent (4–5)	[72,73]
EVA	5–100	20	90	Excellent	Good	Good	Excellent (4–5)	[12,71,74]
PP	600	−18	175	Poor	Good	Excellent	Good (4)	[12,71]
PE	200	−90	137	Poor	Good	Excellent	Good (4)	[12,71]
PVC	2500–3000	90	200	Excellent	Poor	Poor	Poor (3–4)	[75,76,77]
Invisalign	700–900	120	220	Excellent	Good	Good	Good (4)	[39,41,52,58]
Generic multilayer	700–900	120	220	Excellent	Good	Good	Good (4)	[17,18,78]
TC-85 3d resin	2000–2500	75	-	Poor	No data	No data	Poor (3–4)	[22,64,78,79]
Dental LT 3D resin	2000–2500	75	-	Poor	No data	Poor	Poor (3–4)	[64,79]
Accura 60 SLA	2700–3000	60	-	Good	No data	No data	Toxic (1)	[79]
E-Guard Envisiontec 3D	2000–2200	No data	No data	No data	No data	No data	Poor (3–4)	[66,79]

## Data Availability

No new data were created or analyzed in this study.

## Abbreviations

The following abbreviations are used in this manuscript:

CAT	Clear aligner technique
NiTi	Nickel-Titanium superelastic alloy
CAM	Computer-aided manufacturing
SMP	Shape memory polymers
PET	Polyethene terephthalate
PETG	Polyethene terephthalate glycol-modified
TPU	Generic thermoplastic polyurethane
hTPU	High hardness thermoplastic polyurethane
mTPU	Middle hardness thermoplastic polyurethane
sTPU	Soft hardness thermoplastic polyurethane
PCTG	Poly cyclohexylenedimethylene terephthalate glycol-modified Copolyester
PVC	Polyvinyl chloride
EVA	Ethylene vinyl acetate resin
PP	Polypropylene
PC	Polycarbonate
Tg	Glass transition
Tm	Melting point
BPA	Bisphenol A
BPF	Bisphenol F
BPS	Bisphenol S
DMTA	Dynamic mechanical thermal analysis
DSC	Differential scanning calorimetry
3PB	Three-point bending
OMSS	Orthodontic measurement and simulation system
LDH	Lactate dehydrogenase
DIC	Digital image correlation
LCA	Life-cycle assessment
E	Elastic modulus
